# Autophagy through 4EBP1 and AMPK regulates oxidative stress-induced premature senescence in auditory cells

**DOI:** 10.18632/oncotarget.2874

**Published:** 2014-12-30

**Authors:** Nana Akagi Tsuchihashi, Ken Hayashi, Katsuaki Dan, Fumiyuki Goto, Yasuyuki Nomura, Masato Fujioka, Sho Kanzaki, Shizuo Komune, Kaoru Ogawa

**Affiliations:** ^1^ Department of Otorhinolaryngology, Head and Neck Surgery, Keio University, School of Medicine, Tokyo 160–8582, Japan; ^2^ Department of Otorhinolaryngology, Head and Neck Surgery, Kyushu University, School of Medicine, Fukuoka 812–0054, Japan; ^3^ Department of Otorhinolaryngology, Kamio Memorial Hospital, Tokyo 101–0063, Japan; ^4^ Collaborative Research Resources, Core Instrumentation Facility, Keio University, Tokyo 160–8582, Japan; ^5^ Department of Otorhinolaryngology-Head and Neck Surgery, Nihon University, School of Medicine, Tokyo 173–8610, Japan

**Keywords:** premature senescence, autophagy, AMPK, oxidative stress, auditory cell

## Abstract

The aim of this study was to determine whether autophagy and AMPK contribute to premature senescence in auditory cells. Incubating HEI-OC1 auditory cells with 5 mM H_2_O_2_ for 1 h induced senescence, as demonstrated by senescence-associated β-galactosidase (SA-β-gal) staining. H_2_O_2_ treatment significantly delayed population-doubling time, leaving cell viability unchanged. Furthermore, the proportion of SA-β-gal-positive cells significantly increased. Autophagy-related protein expression increased, with Atg7 and LC3-II peaking 6 h and Lamp2 peaking 24 h after H_2_O_2_ treatment. The expression of these proteins decreased 48 h after treatment. Transmission electron microscopy revealed lipofuscin and aggregates within autolysosomes, which accumulated markedly in the cytoplasm of HEI-OC1 cells 48 h after treatment. Akt and P70S6 phosphorylation markedly decreased after H_2_O_2_ treatment, but 4EBP1 phosphorylation significantly increased 48 h after treatment. After RNAi-mediated knockdown (KD) of Atg7 and AMPK, H_2_O_2_-treated cells displayed dense SA-β-gal staining. Also, premature senescence was significantly induced. These suggest that a negative feedback loop may exist between autophagy and AMPK signaling pathways in HEI-OC1 cells. In our model, oxidative stress-induced premature senescence occurred due to impaired autophagy function through 4EBP1 phosphorylation. Our results also indicate that AMPK may regulate premature senescence in auditory cells in an autophagy-dependent and independent manner.

## INTRODUCTION

Aging is a physiological phenomenon that occurs without fail in all eukaryotes. Cellular senescence manifests as stable cell cycle arrest with active metabolism. Cellular senescence could also play a critical role in aging [1].

The study of cellular senescence began in 1961 when Hayflick and Moorhead discovered that human fibroblasts could only divide a finite number of times in culture [2]. This phenomenon, called replicative senescence, was subsequently determined to result from telomere shortening due to repeated chromosome replication and division [3]. Another kind of cellular senescence is stress-induced premature senescence (SIPS), which is early cellular senescence induced by an oncogene [4] or a variety of stressors, such as oxidative stress or chemotherapeutic agents [5]. SIPS occurs irrespective of telomere shortening status. This shows that cellular senescence does not necessarily need to unfold after extended time passage.

Presbycusis or age-related hearing loss (AHL) results as the function of sensory hair cells, spiral ganglion neurons, and stria vascularis cells in the cochlea of the inner ear deteriorates with age [6, 7]. Hearing loss can also result from premature senescence of auditory cells induced by oxidative stress. Oxidative stress induces Bak-dependent mitochondrial apoptosis, which has been demonstrated to be an important mechanism underlying AHL in C57BL/6J mice [8]. How oxidative stress-induced premature senescence specifically affects auditory cellular function remains unclear.

Autophagy is an essential, homeostatic process by which cells break down unnecessary or dysfunctional cellular components. This evolutionarily conserved process is characterized by the formation of double membrane cytosolic vesicles (called autophagosomes), which sequester cytoplasmic content and then fuse with lysosomes [9, 10]. Autophagy enables cells to recycle aggregated proteins and damaged organelles in the cytoplasm that are essential for living cells to recover from stress, such as energy depletion and oxidative and endoplasmic reticulum stress [11–13]. In short, autophagy plays a housekeeping role in cells.

Autophagy dysfunction has been suggested to induce age-related diseases such as Alzheimer's disease [14], cardiomyopathy [15], and hypercholesterolemia [16]. On the other hand, autophagy induction by spermidine or through calorie restriction extends the life span of yeast, flies, worms, mice, and human immune cells [17] [18]. Impairing autophagy through the RNAi-mediated knockdown of autophagy-related genes induces premature senescence in primary human fibroblasts [19], further underscoring the notion that autophagy plays an important role in extending life span. Reducing calorie intake has been shown to delay the progression of AHL [20, 21], but the molecular mechanism underlying oxidative stress-induced autophagy and premature senescence in auditory cells remains unclear.

AMP-activated protein kinase (AMPK) is an evolutionarily conserved energy sensor that controls nutrient sensing and energy homeostasis. AMPK affects many aspects of cellular function, especially cellular senescence [22]. Acute activation of AMPK also prevents H_2_O_2_-induced premature senescence in primary human keratinocytes [23], so AMPK could regulate cellular senescence through oxidative stress. Alternatively, its role in autophagy may affect cellular senescence. AMPK regulates autophagy through two different pathways: one involving mammalian target of rapamycin (mTOR) and the other involving the direct activation of unc-51-like kinase 1 (Ulk1) [24]. Mammalian TOR is a key regulator of autophagy and comprises two major components, mTORC1 and mTORC2 [25]. Despite these known functions, AMPK's role in cellular senescence via autophagy remains elusive in auditory cells.

In light of the above findings, we examined the possible roles of autophagy and AMPK in oxidative stress-induced premature senescence in auditory cells. Conditionally immortalized mouse auditory cells, House Ear Institute-Organ of Corti 1 (HEI-OC1) auditory cells [26], were incubated with a low dose of H_2_O_2_, which induces a senescent phenotype [27]. In addition, we examined how autophagy and AMPK impairment affects replicative life span by using RNAi-mediated knockdown of autophagy-related 7 (Atg7), a mediator of autophagosome biogenesis, and AMPK in auditory cells.

## RESULTS

### Low dose of H_2_O_2_ induces premature senescence in HEI-OC1 cells

**Figure 1:**
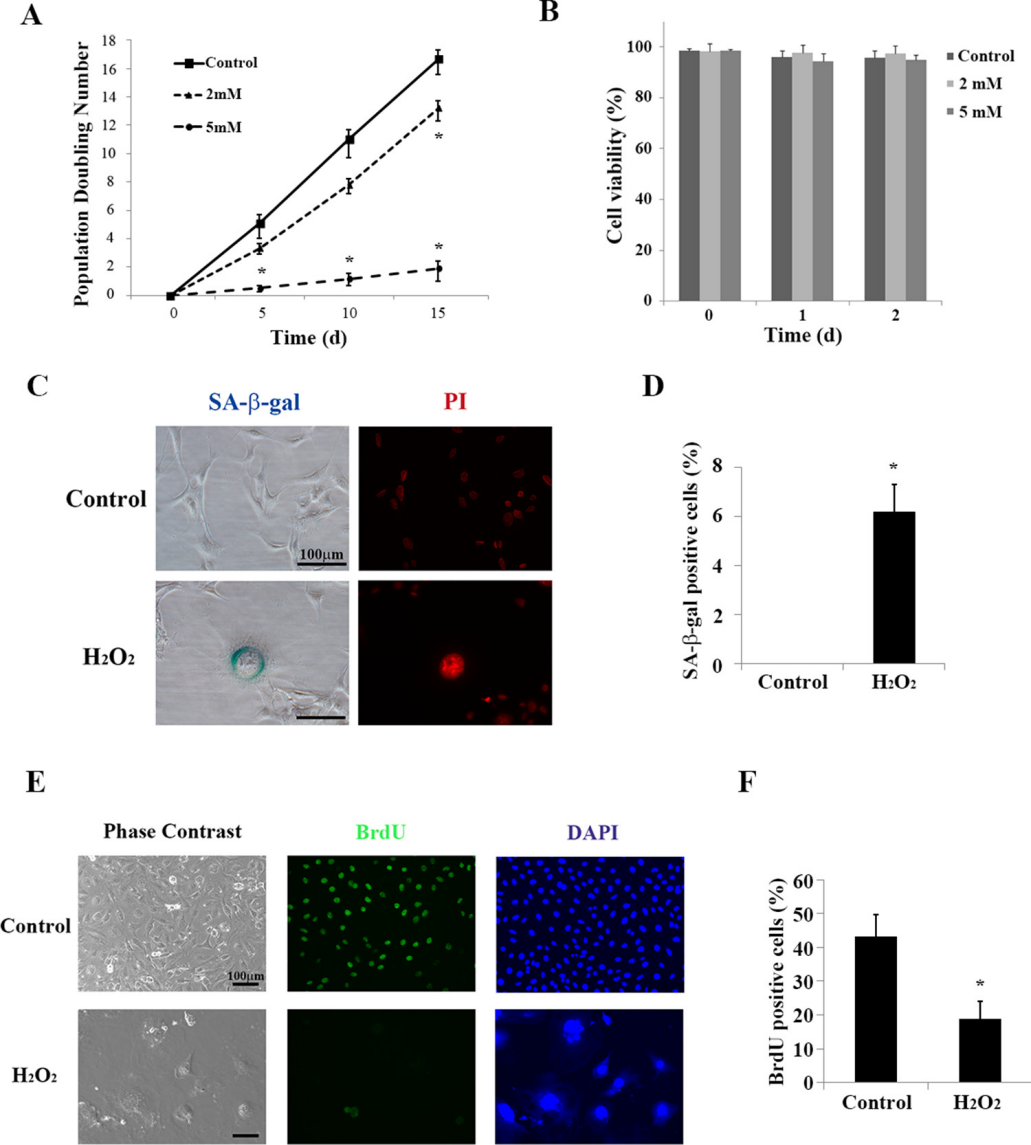
Brief treatment with H_2_O_2_ induces premature senescence in HEI-OC1 cells **(A)** Population doubling time. Population doubling experiments were performed in duplicate, as described in the Methods. **(B)** Cell viability was determined by trypan blue staining at the indicated times after brief (1 h) treatment with H_2_O_2_ (2 mM and 5 mM). All values are means ±S.D. from three or more independent studies. *P < 0.05. **(C)** Representative senescence-associated β-galactosidase (SA-β-gal) staining of HEI-OC1 cells. Propidium iodide (PI) staining labeled nuclear DNA. The assay was carried out in duplicate 2 days after completing the treatment described in the Methods (Cell Viability Assay section). **(D)** SA-β-gal-positive cells were quantified by counting more than 100 cells for each sample. All values are means ±S.D. from three or more independent studies. The control condition exhibited no detectable SA-β-gal staining; *P < 0.05. **(E)** Representative bromodeoxyuridine (BrdU) assay results conducted 2 days after brief treatment with H_2_O_2_ (5 mM for 1 h). DAPI was used to counterstain DNA in the nucleus for cell identification. **(F)** BrdU positive HEI-OC1 cells were quantified by counting more than 100 cells for each sample. Values are means ±S.D. from three or more independent studies. *P < 0.05.

Premature cellular senescence can be induced by applying H_2_O_2_ in a concentration-dependent manner [27]. To determine whether oxidative stress plays a role in cellular senescence in auditory cells, we treated cultured HEI-OC1 cells with different concentrations of H_2_O_2_ (2 mM or 5 mM for 1 h), washed out the H_2_O_2_ with normal culture medium, and then incubated the cells under permissive conditions [26]. The rate of population doubling significantly decreased by 5 days with 5 mM H_2_O_2_, and then by 15 days with 2 mM and 5 mM H_2_O_2_ (Figure 1A). We confirmed that treatment with the 5 mM H_2_O_2_ for 1 h had no effect on cell viability (Figure 1B), while continuous 5 mM H_2_O_2_ treatment significantly decreased viability within 3 to 6 h (data not shown). Since we obtained significant changes in population doubling without affecting cell viability with brief application of H_2_O_2_ (5 mM for 1 h), we selected the same condition for further experiments.

Senescence-associated beta-galactosidase (SA-β-gal) is the most widely known biomarker of cellular senescence [28]. We examined SA-β-gal staining in cultured HEI-OC1 cells treated briefly with H_2_O_2_, as described in the Methods (Cell Viability Assay section). Phase contrast microscopy analysis showed that a significantly increased number of SA-β-gal-positive cells were among the H_2_O_2_-treated cells (0.00 ± 0.00% [control] versus 6.17 ± 1.13% [treated]; *n* = 5, *p* < 0.001) (Figure 1C and 1D). Cells also exhibited marked morphological changes, including increased cell size and change in organelle shape, which corresponds to some of the characteristics of senescent cells [29–32].

We further performed staining with propidium iodide (PI) in treated and control cells to examine the morphology of nuclei. Figure 1C shows that the nuclei lost their sharp outlines under epifluorescence optics, and there were changes in nuclear morphology reminiscent of chromatin condensation 2 days after H_2_O_2_ treatment [33, 34]. PI staining revealed punctuate DNA foci in one large nucleus. This is characteristic of cellular senescence; these foci are termed senescence-associated heterochromatic foci (SAHF) [35].

To examine whether cell proliferation is attenuated under oxidative stress, we incorporated bromodeoxyuridine (BrdU) into cultured HEI-OC1 cells. BrdU can be incorporated into the newly synthesized DNA of replicating cells during the S phase of the cell cycle. The proportion of cells incorporating BrdU significantly decreased 2 days after the brief H_2_O_2_ treatment (43.11 ± 6.5% [control] versus 18.29 ± 5.07% [5 mM H_2_O_2_ for 1 h], *n* = 5, *p* < 0.001) (Figure 1E and 1F). These findings indicate that a brief treatment of H_2_O_2_ induces premature senescence in HEI-OC1 cells without leading to cell death.

### H_2_O_2_ treatment induces autophagy in HEI-OC1 cells

**Figure 2:**
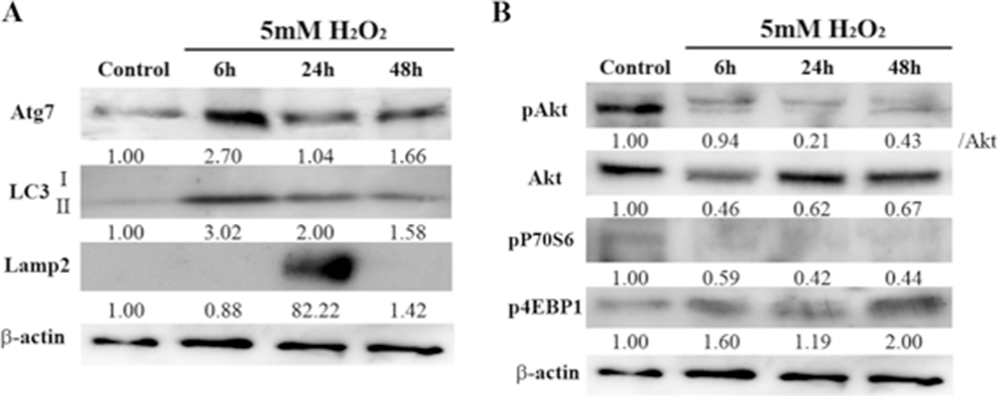
Effects of brief H_2_O_2_ treatment on autophagy signaling pathway in HEI-OC1 cells **(A)** Representative Western blots showing the expression of autophagy-related 7 (Atg7), macrotubule-associated protein 1 light chain 3 (LC3), and lysosome-associated membrane protein 2 (Lamp2) in control and H_2_O_2_-treated cells. β-actin was included as a loading control. The optical density of Atg7, LC3 II, and Lamp2 in H_2_O_2_-treated cells was normalized to that of the corresponding proteins in control cells, and the resulting ratios are indicated below each blot. **(B)** Western blot analysis of the mammalian target of rapamycin (mTOR) pathway, which negatively regulates autophagy. This pathway includes phosphorylated Akt (pAkt), Akt, pP70S6, and p4EBP1. β-actin was included as a loading control. The optical density of pAkt, Akt, pP70S6, and p4EBP1 in H_2_O_2_-treated cells were normalized to that of the corresponding proteins in control cells, and the resulting ratios are indicated below each blot.

Because autophagy plays an important role in mediating cell survival in response to various stressor stimuli, including oxidative stress [36–38], and because it can be regulated by H_2_O_2_ [39], we examined the induction of autophagy in HEI-OC1 cells treated with a low dose of H_2_O_2_. As shown in Figure 2A, Atg7 and macrotubule-associated protein 1 light chain 3-II (LC3-II) expression levels significantly increased, peaking 6 h after H_2_O_2_ treatment, followed by lysosome-associated membrane protein 2 (Lamp2) activation, which peaked at 24 h. However, the expression of these proteins (Atg7, LC3-II, Lamp2) decreased 48 h after treatment, indicating that, under these brief H_2_O_2_ conditions, autophagy was impaired at 48 h.

To elucidate in detail the autophagic pathway induced by the H_2_O_2_ stressor in auditory cells, we further evaluated the mTOR cascade. Mammalian TOR is a multidomain protein kinase that interacts with other proteins to form two main complexes, mTORC1 and mTORC2. Mammalian TORC1 signaling impairs autophagy [9]. Akt is an upstream regulator of mTORC1 and an effector of mTORC2, whereas S6Ks and 4EBPs are downstream substrates of mTORC1 [40]. H_2_O_2_ treatment markedly reduced Akt phosphorylation, but Akt expression remained the same. Phosphorylation of P70S6 kinases (pP70S6) significantly decreased after brief treatment with H_2_O_2_ phosphorylation of 4E-binding protein 1 (p4EBP1) increased 48 h after treatment (Figure 2B). Taken together, these results support the idea that Akt activity regulates only the phosphorylation of S6K1 but not 4EBP1 in auditory cells.

### Ultrastructural changes in the autophagic structures of HEI-OC1 cells treated with a brief, low dose of H_2_O_2_

**Figure 3:**
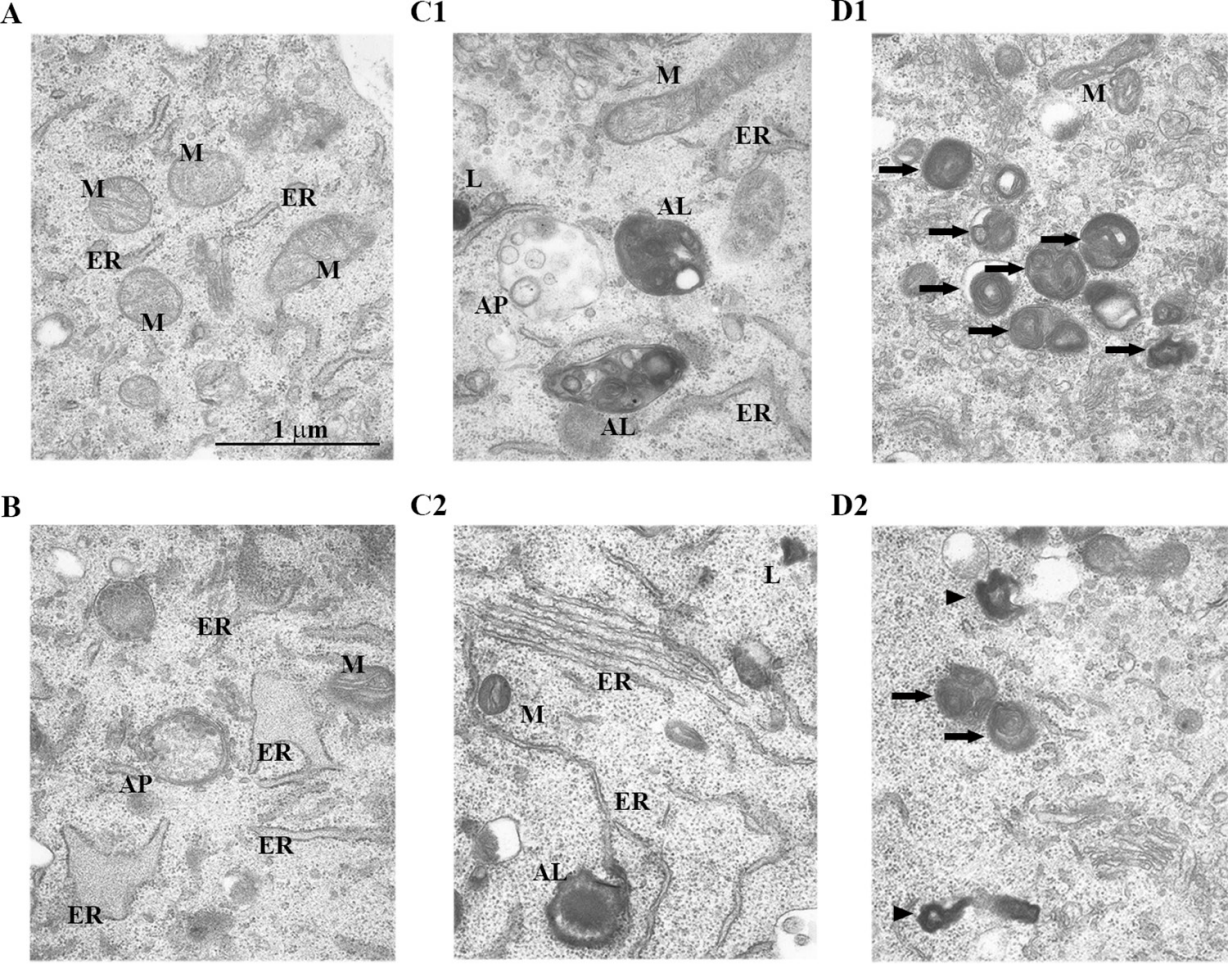
Changes in the ultrastructure of autophagic structures in HEI-OC1 cells treated with H_2_O_2_ briefly Representative transmission electron microscopy (TEM) photomicrographs of HEI-OC1 cells. Cells were treated as in cell viability and population doubling experiments (Methods). **(A)** Control cell. **(B–D)** Cells 6 h (B), 24 h (C1, C2), and 48 h (D1, D2) after treatment with 5 mM H_2_O_2_ for 1 h. AP, autophagosome; AL, autolysosome; M, mitochondria; ER, endoplasmic reticulum; L, lysosome. The arrowheads and arrows point to lipofuscin and aggregates, respectively.

We examined ultrastructural autophagic processes in HEI-OCI cells treated with a brief, low dose of H_2_O_2_. Transmission electron microscopy (TEM) revealed that cells in the control condition exhibited normal nuclei with uniform and finely dispersed chromatin. Cytoplasm in these cells also had normal appearing mitochondria (M) and endoplasmic reticulum (ER) (Figure 3A). By contrast, H_2_O_2_-treated cells had accumulated a small number of autophagic vacuoles 6 h after treatment. At this time, autophagosomes were round, double-membraned structures, containing electron lucent material and/or dense organelles (Figure 3B). Twenty-four hours after treatment, some autophagosomes were round or oval double-membraned structures, containing the same contents as autophagosomes examined in cells prepared 6 h after treatment. Other autophagosomes eventually appeared to merge with lysosomes to become autolysosomes containing partially degraded material that appeared as electron dense, unevenly distributed dense masses (Figure 3C1). At this time, autophagosomes and autolysosomes had accumulated to higher concentrations in the treated cells. Figure 3C2 shows an autophagosome that appeared to form at some ER and became an autolysosome that enveloped damaged mitochondria. Forty-eight hours after treatment, intact lipofuscin and aggregates within autolysosomes accumulated markedly in the cytoplasm [41] (Figure 3D1 and 3D2). These observations support the hypothesis that a briefly applied H_2_O_2_ impairs autophagic processes at the ultrastructural level, appearing as soon as 48 h after H_2_O_2_ treatment.

### Impaired autophagy induces premature senescence in HEI-OC1 cells

**Figure 4:**
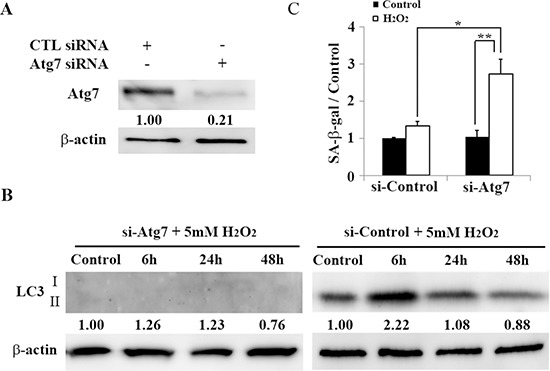
Atg7-knockdown associated autophagy impairment induces premature senescence in HEI-OC1 cells **(A)** Western blots of lysates from HEI-OC1 cells knocked down with *Atg7* siRNA (see Methods). β-actin was included as a loading control. **(B)** Western blots of lysates from H_2_O_2_-treated HEI-OC1 cells. After transfection with *Atg7* siRNA for 2 days, cells were treated with H_2_O_2_ (5 mM, 1 h), and then cultured for 2 days before harvesting and preparation for Western blotting analyses. β-actin was included as a loading control. The optical density of LC3 II in H_2_O_2_-treated cells were normalized to that of LC3 II proteins in control cells, and the resulting ratios are indicated below each blot. **(C)** Quantification of SA-β-gal staining intensity 2 days after treatment with 5 mM H_2_O_2_ (1 h) and transfection with control or *Atg7* siRNA. Quantitative fluorescence analysis was conducted as described in the Methods. All values are means ± S.D. from three or more independent studies. *P < 0.05; **P < 0.01.

We next examined the relationship between premature senescence and autophagy in HEI-OC1 cells, which were induced by treatment with brief application of H_2_O_2_. When *Atg7* was knocked down by siRNA (Figure 4A), autophagy was successfully inhibited and LC3 levels were attenuated, as assessed by Western blotting (Figure 4B). Expression of SA-β-gal was significantly elevated in Atg7-depleted HEI-OC1 cells 2 days after H_2_O_2_ treatment (1.34 ± 0.19 [control] versus 2.72 ± 0.39 [5 mM H_2_O_2_ for 1 h]; *n* = 3, *p* < 0.02), indicating its dependence on autophagy (Figure 4C). These results suggest that impairing autophagy induces premature senescence in auditory cells, as assessed by SA-β-gal.

### AMPK participates in the induction of premature senescence in HEI-OC1 cells

**Figure 5:**
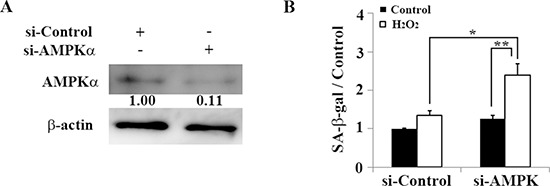
AMPK dysfunction resulting from AMPKα knockdown leads to premature senescence in HEI-OC1 cells **(A)** Representative Western blot showing AMPKα expression in HEI-OC1 cells transfected with control or *AMPKα* siRNA. AMPKα expression levels were determined 2 days after transfection to validate the efficiency of *AMPKα* knockdown. β-actin was included as a loading control. The optical density of AMPKα in H_2_O_2_-treated cells was normalized to that of AMPKα in si-control cells, and the resulting ratios are indicated below each blot. **(B)** Quantification of SA-β-gal staining intensity 2 days after 1-h treatment with 5 mM H_2_O_2_ and transfection with control siRNA or *AMPKα* siRNA. Quantitative fluorescence analysis was conducted as described in the Methods. All values are means ±S.D. from three or more independent studies. *P < 0.05; **P < 0.01.

We further examined how AMPK contributes to premature senescence through oxidative stress in auditory cells. In HEI-OC1 cells knocked down with AMPKα siRNA (Figure 5A), SA-β-gal significantly increased 2 days after H_2_O_2_ treatment (1.34 ± 0.12 [control] versus 2.39 ± 0.31 [5 mM H_2_O_2_]; *n* = 3, *p* < 0.04). This indicates that induction of premature senescence in auditory cells depends not only on autophagy impairment but also on AMPK dysfunction (Figure 5B).

### A negative feedback loop exists between autophagy and AMPK signaling pathways in HEI-OC1 cells

**Figure 6:**
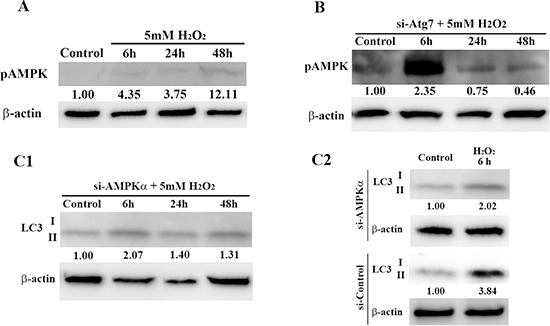
Interaction between autophagy and AMPK signaling pathways in HEI-OC1 cells **(A)** Western blot analysis of AMPK phosphorylation (pAMPK) in control cells and H_2_O_2_-treated cells. **(B)** Representative Western blot showing pAMPK expression at the indicated times after H_2_O_2_ treatment. HEI-OC1 cells were transfected with *Atg7* siRNA, treated with 5 mM H_2_O_2_ for 1 h, and then pAMPK expression levels were determined by Western blot 2 days later. β-actin was included as a loading control. **(C1, C2)** Western blots of lysates from HEI-OC1 cells transfected with *AMPKα* siRNA. As above, LC3 expression was assessed after H_2_O_2_ treatment (5 mM, 1 h) and 2 days after transfection. β-actin was included as a loading control. LC3-II expression in H_2_O_2_-treated AMPKα KD cells was lower than that in si-control cells 6 h after H_2_O_2_ treatment. For all blots (A-C), the optical density of the indicated proteins in H_2_O_2_-treated cells was normalized to that in control cells; the resulting ratios are indicated below each respective blot. All values are means ± S.D. from three or more independent studies.

To better understand the relationship between autophagy and AMPK in H_2_O_2_-induced premature senescence in HEI-OC1 cells, we assessed AMPK phosphorylation in H_2_O_2_-treated si-control cells and Atg7 knockdown (KD) cells, and LC3 expression in si-control cells and AMPKα KD cells. Figure 6A shows that brief treatment with H_2_O_2_ led to phosphorylation of AMPK in a time-dependent manner. Knockdown of *Atg7* resulted in robust phosphorylation of AMPK, which occurred rapidly 6 h after H_2_O_2_ treatment (Figure 6B). However, RNAi-mediated knockdown of *AMPKα* decreased LC3-II expression relative to expression in si-control cells 6 h after treatment (5 mM, 1 h) (Figure 6C1, 6C2). These results support the existence of a negative feedback loop between autophagy and AMPK signaling pathways in auditory cells.

## DISCUSSION

In this study, we demonstrated that oxidative stress-induced premature senescence in auditory cells is regulated by autophagy through 4EBP1 and AMPK. Several lines of evidence support this conclusion. Briefly applied H_2_O_2_ at a low dose (5 mM for 1 h) induced premature senescence in HEI-OC1 auditory cells *in vitro*. Autophagy was detected 24 h after the brief H_2_O_2_ treatment and was impaired 48 h after treatment. During this time, lipofuscin and subcellular aggregates markedly accumulated in the cytoplasm of these cells. Phosphorylation of AKt and P70S6 decreased in a time-dependent manner, while phosphorylation of 4EBP1 increased 48 h after H_2_O_2_ treatment. Autophagy impairment and AMPK dysfunction led to premature senescence in these auditory cells. Taken together, these findings provide evidence that a negative feedback loop is present in auditory cells that includes autophagy and AMPK signaling pathways.

To establish an auditory cell model of premature senescence, we used low doses of H_2_O_2_ as a stressor to induce premature senescence in HEI-OC1 auditory cells. H_2_O_2_ has been used to induce premature senescence in other cell types as well, such as vascular endothelial cells [42] and keratinocytes [24]. In our system, brief H_2_O_2_ treatment resulted in delayed cell proliferation in the absence of a remarkable decrease in cell viability (Figure 1A and 1B), suggesting that the cells underwent oxidative stress-induced premature senescence. As shown in Figure 1C, the cytoplasm of cells displaying morphological characteristics common to cellular senescence stained intensely for SA-β-gal, a distinct marker of cellular senescence. By contrast, the proportion of BrdU incorporated into newly synthesized DNA significantly decreased. These results support the use of our system as a model for studying premature senescence in auditory cells. This report is the first to show that SA-β-gal is useful for staining non-dividing, postmitotic cells like auditory cells.

In this study, we also confirmed that brief application of H_2_O_2_ induced Atg7 expression, which peaked 6 h after treatment (Figure 2). This correlated well with the expression of LC3-II in auditory cells (Figure 2). We chose to assess Atg7, a mediator of autophagosomal biogenesis, because it has been demonstrated to be a viable target through which to activate basal autophagic function in another cell type, cardiomyocytes [43]. Moreover, genetic deletion of Atg7 results in an overt loss of autophagy, showing that Atg7 is necessary for processing LC3-I to activate LC3-II and autophagosome formation [44]. Our results suggest that autophagy is induced at an early point in oxidative stress-induced premature senescence, and Atg7 is important for autophagosome formation in auditory cells and the subsequent induction of cellular senescence.

Lamp2 induction peaked 24 h after H_2_O_2_ treatment. Lamp2 is required for the proper fusion of lysosomes with autophagosomes in the last stages of the autophagic process [45]. This suggests that in auditory cells, autophagosomes fuse with lysosomes, and then become autolysosomes within 24 h. Interestingly, the expression of Atg7, LC3-II, and Lamp2 decreased 48 h after induction with H_2_O_2_, indicating that autophagy function had declined by this time. In fact, TEM revealed that a few autophagosomes already exist in the cytoplasm as early as 6 h after treatment with low doses of H_2_O_2_. The presence of autolysosomes was confirmed 24 h after treatment.

**Figure 7:**
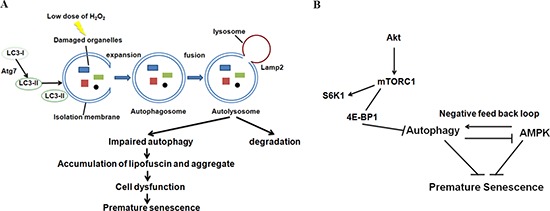
Autophagy and AMPK play important roles in the regulation of oxidative stress-induced premature senescence in auditory cells Hypothesized schematic model. **(A)** Autophagy impairment leads to the accumulation of lipofuscin and aggregates in cytoplasm, ultimately resulting in auditory cell dysfunction. Our results indicate that premature senescence was induced in our auditory cellular senescence model. **(B)** The regulation of autophagy through 4EBP1 phosphorylation plays a key role in oxidative stress-induced premature senescence of auditory cells. In addition, autophagy-dependent and independent involvement of AMPK is pivotal for regulating premature senescence in auditory cells.

Also, lipofuscin and subcellular aggregates, which accumulate heavily during aging and appear to affect cellular function [41], increased in number in the cytoplasm 48 h after H2O2 treatment in our model system. This clearly indicates that autophagy was induced by 24 h after H2O2 treatment and was impaired 48 h after treatment. It is reasonable to propose that auditory cells progressively became dysfunctional due to the accumulation of lipofuscin and aggregates in the cytoplasm, reaching premature senescence at the 48-h point in our model (Figure 7A).

We confirmed that Akt phosphorylation was significantly decreased in our model. Low levels of Akt phosphorylation are associated with stress-induced senescence in endothelial cells [46]. It is well known that Akt is a key positive regulator of mTORC1, a negative regulator of autophagy. As shown in Figure 2B, P70S6 phosphorylation decreased in proportion to that of Akt in a time-dependent manner, whereas 4EBP1 phosphorylation (p4EBP1) increased independent of the Akt-mTORC1 pathway 48 h after H_2_O_2_ treatment. These results indicate that Akt activity regulates only S6K1 in auditory cells. Considering that in our model premature senescence was induced 48 h after H_2_O_2_ treatment resulting in autophagy impairment, the regulation of autophagy depends on 4EBP1. Indeed, a recent study demonstrated a possible mTORC1-independent role for 4EBP1 in the regulation of senescence in another model [47].

Many studies have shown that autophagy activation mediates the extension of life span [18, 48]. Conversely, it has been reported that autophagy impairment induces premature senescence in primary human fibroblasts [49]. In the present study, RNAi-mediated knockdown of *Atg7* efficiently led to autophagy impairment in auditory cells. There were a significant proportion of senescent cells among H_2_O_2_-treated Atg7 KD cells (Figure 4C). These findings indicate that autophagy impairment due to decreased Atg7 expression may be responsible for premature senescence in auditory cells. To our knowledge, this is the first study to demonstrate that reduction of Atg7 induces premature senescence in auditory cells.

We also investigated the effects of AMPK on H_2_O_2_-induced premature senescence in auditory cells, because AMPK regulates cell survival during stress and coordinates signaling of many age-related transcription factor pathways. A recent study indicates that acute activation of AMPK prevents H_2_O_2_-induced premature senescence in primary human keratinocytes [24]. In our present study, as expected, there was a significantly increased number of densely SA-β-gal stained senescent cells among the H_2_O_2_-treated Atg7 KD cells (Figure 5B). AMPK activation upregulates antioxidant mechanisms that diminish H_2_O_2_ [50], while AMPK knockdown increases oxidative stress in 3T3L1 adipocytes [51]. Therefore, the increased number of SA-β-gal positive cells we observed after AMPK KD could be due to oxidative stress. This suggests that another possible mechanism may exist in auditory cells for AMPK regulation of premature senescence through oxidative stress. Indeed, we observed that AMPK phosphorylation (i.e., AMPK activation) increased in a time-dependent manner, reaching a maximum 48 h after H_2_O_2_ treatment. This finding indicates that, in turn, AMPK activation upregulates antioxidant mechanisms, since premature senescence was reliably observable in our model 48 h after brief H_2_O_2_ treatment. Therefore, these apparently conflicting findings indicate that the mechanism in auditory cells—premature senescence regulation through AMPK—could involve regulation of oxidative stress.

AMPK also plays a role as a key activator of the signaling network that maintains cellular housekeeping by autophagy [52, 53]. AMPK usually stimulates autophagy by inhibiting mTOR, but also by directly phosphorylating ULK1 (mammalian homolog of yeast and fly Atg1) [54, 55]. In fact, RNAi-mediated knockdown of *AMPKα* reduced the expression of LC3-II to less than si-control. However, knockdown of Atg7 leads to the early induction of pAMPK 6 h after treatment. This result indicates AMPK activity could be negatively regulated by Atg7 in auditory cells. Namely, there is a negative feedback loop between autophagy and AMPK pathways in auditory cells (Figure 7B).

In summary, we confirmed that impaired autophagic processes induce oxidative stress-related premature senescence in our auditory cellular premature senescence model. Autophagy was impaired through the phosphorylation of 4EBP1. We also found that autophagy impairment and AMPK dysfunction induced premature senescence in auditory cells. Taken together, these results indicate that AMPK regulates premature senescence in an autophagy-dependent and independent manner. Our results provide significant insights into the role of autophagy (thorough 4EBP1 and AMPK) in oxidative stress-induced premature senescence of auditory cells. To our knowledge, this is the first study to show the association between premature senescence and autophagy in auditory cells. However, further studies need to be conducted to determine how senescence and autophagy affect auditory cellular function and contribute to pathology underlying hearing impairments.

## METHODS

### Regents and antibodies

Hydrogen peroxide was purchased from Wako (Wako, Osaka, Japan). The following primary antibodies were purchased from Cell signaling Technology (Danvers, MA, USA): anti-Atg7, anti-pP70S6, anti-p4EBP1, anti-AMPKα, anti-pAMPKα. Anti-LC3, anti-Lamp2, and anti-β-actin antibodies were obtained from Sigma-Aldrich (St. Louis, MO, USA); Santa Cruz Biotechnology, Inc. (Dallas, TX, USA); and BD Biosciences (San Jose, CA, USA), respectively. Anti-mouse and –rabbit HRP-linked secondary antibodies were from GE Healthcare (Buckinghamshire, UK). Small interfering RNA (siRNA) for *Atg7*, *AMPK*, and control siRNA were from Cell Signaling Technology. Propidium iodide (PI) solution was also from Cell Signaling Technology. Bromodeoxyuridine (BrdU) and anti-BrdU (Bu20a) antibody were purchased from Cell Signaling Technology. FITC-conjugated secondary antibody was from Life Technologies (Carlsbad, CA, USA).

### Cell culture and culture conditions

HEI-OC1 auditory cells were kindly provided by Dr. D. Lim (House Research Institute, Los Angeles, CA, USA). This conditionally immortalized mouse auditory cell line was established by Kalinec et al. [27], and is a well-established *in vitro* system for investigating cellular and molecular mechanisms. Cells were maintained in low-glucose Dulbecco's modified medium (DMEM; Wako, Osaka, Japan) supplemented with 10% fetal bovine serum (FBS; HyClone, UT, USA), 1% penicillin streptomycin, and 1% kanamycin (MP Biomedicals, Ohio, USA) at 33°C in a humidified incubator with 10% CO_2_. This culturing medium also served as the control medium in the population doubling experiments. Maintaining HEI-OC1 cells at this temperature and CO_2_ concentration is referred to as “permissive conditions” by Kalinec et al. [27], because lower basal apoptotic rates are present.

### Transient siRNA transfection

Small interfering RNA (siRNA) against *Atg7* (Sigma-Aldrich), *AMPK1/2* (Santa Cruz Biotechnology, Inc.), and control siRNA (Cell Signaling Technology) were used for knockdown of the *Atg7* and *AMPK1/2* genes. HEI-OC1 cells were seeded in 25 cm^2^ flasks and cultured overnight to achieve 90–95% confluency. Cells were then mechanically scraped from the flasks and transfected with siRNAs using a Gene Pulser electroporation system (Bio-Rad, Hercules, CA, USA), according to the manufacturer's protocol. Two days after transfection, cells were either treated with H_2_O_2_ or remained untreated and then prepared for Western blot analysis (see below).

### Cell viability assay

HEI-OC1 auditory cells (5 × 10^4^ cells/ml/well of 24-well plates) were incubated with one of two different concentrations of H_2_O_2_ (2 or 5 mM), dissolved in the control medium listed in the cell culture and culture condition section for 1 h, and then the H_2_O_2_ solution was replaced with culture medium lacking H_2_O_2_. Control cells were also prepared similarly except without H_2_O_2_. To determine viability, the cells were washed with phosphate-buffered saline (PBS), harvested from the flasks via trypsinization (0.25w/v% trypsin-1 mmol/l EDTA, for 2 min.), resuspended in PBS, and diluted 1:1 in 0.4% trypan blue solution. Cell viability was calculated with a Countess® Automated Cell Counter (Invitrogen, Life Technologies, USA), following the manufacturer's suggested procedures.

### Measurement of population doublings

To determine the rate of population doublings in HEI-OC1 cells, we used a slightly modified method, as previously described [28]. Cell numbers were counted at the indicated intervals (2 or 3 days for control and 2 mM-H_2_O_2_, and 5 days for 5 mM-H_2_O_2_ group) using a hemocytometer, and then the cells were seeded again in preparation for the next count. The numbers were converted into population doublings according to the following formula: [log (No. of cells counted) - log (No. of cell plated)]/log (2).

### Senescence-associated β-galactosidase (SA-β-gal) staining

SA-β-gal staining in HEI-OC1 cells was performed using a Senescence Cells Histochemical Staining Kit® (Sigma-Aldrich), following the manufacturer's instructions. As above, HEI-OC1 cells were incubated with 5 mM H_2_O_2_ for 1 h, and then the H_2_O_2_ solution was replaced with culture medium lacking H_2_O_2_. Cultured cells were washed with PBS, fixed with Fixative Buffer for 6–7 min, washed again with PBS, and then incubated with the kit's Staining Mixture overnight at 37°C in a humidified incubator without the added 10% CO_2_ mixture (i.e., 100% ambient room air). Then undiluted PI solution was added for staining DNA contents in nuclei. Cells were observed with an inverted fluorescence phase-contrast microscope (BioZero BZ-8100 All-In-One Fluorescence Microscope, Keyence, Osaka, Japan). Cells with blue staining in the cytoplasm were scored as positive. To quantify positive staining, more than 100 cells were counted for each sample in duplicate experiments.

Quantitative analysis of fluorescent SA-β-gal staining was carried out using a 96-well Cellular Senescence Assay Kit® (Cell Biolabs, Inc., San Diego, CA, USA). First, we briefly washed the cells with 1 × PBS and added 1X lysis buffer. After centrifuging the cell lysate for 10 min, the supernatant was collected, and the total protein concentration of each cell lysate sample was determined using a Nanodrop® spectrophotometer (Model #1000, Nanodrop Technologies, DE, USA). The lysate was mixed with the manufacturer's Assay buffer containing 5% SA-β-gal solution, and then incubated at 37°C protected from ambient room light for 1.5 h. The reaction was stopped by adding the manufacturer's Stop solution. SA-β-gal fluorescence was detected and measured with a fluorescence plate reader at 360 nm (excitation wavelength) / 465 nm (emission wavelength).

### BrdU incorporation and detection

HEI-OC1 cells were seeded onto 35 mm dishes, and BrdU incorporation was conducted by adding diluted BrdU in warmed culture medium to a final concentration of 0.03 mg/ml and incubated for 30 min. After aspirating this media, cells were fixed by covering the cell layer with 70% ethanol for 5 min, thoroughly washed in PBS, and then incubated in 1.5 M HCl for 30 min. Cells were then incubated in a mixture of 5% (wt/vol) normal goat serum and 0.3% Triton X-100 in PBS for 1 h, and then incubated overnight at 4°C in anti-BrdU primary antibody (1:1400) diluted in PBS and 1% BSA. After washing thoroughly in PBS, cells were incubated in FITC-conjugated secondary antibody for 2 h in the dark and counterstained with 4′,6-diamidino-2-phenylindole (DAPI). The cells were washed thoroughly in PBS and observed with an all-in-one epifluorescence microscope (BioZero BZ-8100, Keyence). Cell counts were done at 60 × magnification. Cells with positive nuclear staining were scored as positively incorporating BrdU. To quantify positive staining, more than 100 cells were counted for each sample in duplicate.

### Protein extraction and Western blotting

Western blotting was performed as follows. HEI-OC1 cell samples were trypsinized for 2 min and homogenized in 20 mM HEPES, containing 1% Triton X-100. Once homogenized, cell extracts were centrifuged at 10,000 rpm at 4°C and supernatants were collected. The protein concentration of the supernatant was evaluated with a Nanodrop® spectrophotometer (Nanodrop Technologies). An equal volume of 2 × SDS sample buffer was added to the samples, and the samples were then boiled for 3 min. Samples (25 μg) were subjected to electrophoresis on 10–12.5% SDS-polyacrylamide gels for 60–80 min at 40mA and then transferred onto PVDF membranes using iBlot® (Invitrogen). The membranes were incubated overnight at 4°C in 5% (wt/vol) dried milk protein or 2% (wt/vol) bovine serum albumin in TBS containing 0.1% (vol/vol) Tween-20 (TBS-T), washed in TBS-T, and then incubated overnight at 4°C in the presence of primary antibodies (see Reagents and Antibodies section) at dilutions of 1:200–1:1000 in Can Get Signal® solution (TOYOBO, Japan). After 3 washes with TBS-T, the membranes were incubated with the corresponding species-appropriate secondary antibody at a dilution of 1:5000 in Can Get Signal® solution, and developed with Immunostar (Wako, Osaka, Japan).

### Transmission electron microscopy

HEI-OC1 cells were harvested by trypsinization, and then were fixed with 2% glutaraldehyde in 0.1 M sodium cacodylate buffer (pH 7.2) for 1 h followed by 1% osmium tetroxide in 0.1 M sodium cacodylate buffer (pH 7.2) for 2 h. To enhance contrast for TEM, samples were en-bloc stained with 0.5% aqueous uranyl acetate overnight and then dehydrated by alcohols at 4°C and infiltrated with a graded series of Epon/Araldite mixture, followed by embedding in 100% Epon/Araldite. Thin sections (70 nM) were cut with a diamond knife, mounted on EM grids, and stained with Reynolds' lead citrate solution, washed, dried, and imaged using a FEI Tecnai 12 transmission electron microscope (Field Emission Incorporated, Hillsboro, Oregon, U.S.A.).

### Statistical analysis

All data were expressed as means ±S.D. Statistical analysis was carried out by using one-way analysis of variance and Student's *t* tests. *P* values less than 0.05 were considered to indicate statistical significance.
